# Evaluation of the effect of supervised anti-malarial treatment on recurrences of *Plasmodium vivax* malaria

**DOI:** 10.1186/s12936-021-03793-0

**Published:** 2021-06-13

**Authors:** Kelry Mazurega Oliveira Dinelly, Sheila Vitor-Silva, Jose Diego Brito-Sousa, Vanderson Souza Sampaio, Milena Gabriela Oliveira Silva, André Machado Siqueira, Cássio Peterka, Sheila Rodovalho, Aretha Gomes Omena, Wuelton Marcelo Monteiro, Marcus Vinícius Guimarães Lacerda, Gisely Cardoso Melo

**Affiliations:** 1 Programa de Pós-graduação em Medicina Tropical, Universidade do Estado do Amazonas, Manaus, Brazil; 2 Fundação de Medicina Tropical Dr. Heitor Vieira Dourado, Manaus, Brazil; 3 Faculdade Metropolitana de Manaus–FAMETRO, Manaus, Brazil; 4 Escola de Enfermagem de Manaus, Universidade Federal do Amazonas, Manaus, Brazil; 5 Fundação de Vigilância em Saúde do Amazonas–FVS/AM, Manaus, Brazil; 6 Instituto Nacional de Infectologia Evandro Chagas–INI, Fundação Oswaldo Cruz, Rio de Janeiro, Brazil; 7 Instituto de Pesquisa Leônidas & Maria Deane, Fundação Oswaldo Cruz, Manaus, Brazil; 8 Organização Pan Americana da Saúde, Brasília, Brazil

**Keywords:** Malaria, *Plasmodium vivax*, Supervised treatment, Unsupervised treatment, Recurrence

## Abstract

**Background:**

Relapses in vivax malaria have posed great challenges for malaria control, and they also account for a great proportion of reported cases. Knowing the real effectiveness of a 7-day primaquine (PQ) scheme is crucial in order to evaluate not only the cost-effectiveness of implementing new anti-hypnozoite drugs, but also how health education strategies can guarantee better compliance and be reinforced. This study aimed to evaluate the effect of daily treatment with chloroquine and PQ supervised by health workers *versus* prescription without supervision.

**Methods:**

The outcome was the passive detection of new positive thick blood smears up to 180 days, based on the official data records from the National Malaria Control Programme. The recurrences seen in the real life were, therefore, used as a surrogate for true relapses.

**Results:**

Patients under supervised treatment had a lower risk of recurrence up to day 180 when compared to the unsupervised treatment (17.9% *vs.* 36.1%; *p *= 0.027).

**Conclusions:**

The lack of supervision in the non-supervised group (which followed standard of care in the real life) enabled proper comparison, as consent itself would have lead to greater compliance in this group. Future studies should scale such an analysis to different settings in the Brazilian Amazon.

## Background

*Plasmodium vivax* is the most prevalent etiological agent of malaria in Brazil (~ 90%) [1]. The development of latent hepatic forms known as hypnozoites, which are responsible for relapses months or years after an episode of vivax malaria, contribute to the maintenance of the transmission cycle [2]. In 2009, the frequency of recurrences in the Brazilian Amazon was 20.8% while in the municipality of Porto Velho, it was 23% for the same period [3]. Other studies have demonstrated that 30.9% [4] and 29.4% [5] of the individuals in the Brazilian Amazon who were tested presented with malaria recurrence. The Brazilian Ministry of Health recommends chloroquine (CQ) for 3 days and primaquine (PQ) (0.5 mg/kg/day) for 7 days for uncomplicated *P. vivax* malaria. One of the limiting aspects of treatment success is the variation in the response of parasites to the therapeutic regimens used and poor adherence to treatment [6].

The interruption of medication by the patient and difficulty in accessing basic health units are some of the factors that are related to non-adherence and consequently to recurrences [7–9]. Some studies indicate that after the first doses of anti-malarials, patients infected with *P. vivax* become asymptomatic and tend to interrupt treatment with PQ, which is the essential drug for radical cure [10]. To determine the effectiveness of PQ in endemic areas, it is necessary to assess recurrences for up to 180 days, which is enough to detect most recurrences in Brazil [11]. In a rural area in the Western Brazilian Amazon, *P. vivax* relapse episodes were observed in 29.4% of the individuals during 90 days of follow-up [12]. Recurrences of vivax malaria are cause for concern since they are a source of disease transmission, and as such are one of the obstacles to the elimination of malaria. The debate regarding the distinction between recrudescence, relapse and reinfection usually needs biomolecular markers to distinguish them, but even with these tools, a clear distinction has limitations [13]. Thus, this study evaluated the impact supervised treatment has on recurrences of vivax malaria in a municipality in the Brazilian Amazon.

## Methods

Patients over 16 years old, with a positive thick blood smear for vivax malaria, were recruited in malaria diagnostic centres (primary care units) in Rio Preto da Eva, (75 km from the state capital Manaus) Amazonas, Brazil. According to the Brazilian guidelines for treatment of malaria, patients should receive CQ (25 mg/kg for the first 3 days) and a short course of PQ (0.5 mg/kg/day, for 7 days) [1]. All patients with confirmed vivax malaria are prescribed both drugs, without previous G6PD screening, except children under 6 months of age and pregnant women. The treatment for malaria in the Brazilian Amazon is in accordance with patient’s weight and the municipal health professionals were all trained to give doses accordingly. Although patient weight and total dose per kg are important variables and the patients from the unsupervised group were also given the weight-adjusted dose. In Brazil, anti-malarial drugs are given free to patients, and health professionals do not normally supervise their daily administration.

Patients were randomized for consent using Zelen’s design [1], and only one group was randomized to sign an informed consent form (ICF) and was visited daily at home for drug supervision by one health agent. A blocked randomization list and sequential envelopes containing randomizations for unsupervised and supervised medication was prepared by an independent statistician using the statistical package R. Sealed envelopes were provided to the malaria diagnostic centre and were used to randomize patients. Other patients diagnosed with vivax malaria were treated without supervision and without any type of intervention. During the follow-up period, there were no scheduled visits to the supervised or unsupervised group. Follow-up was exclusively via the SIVEP Malaria notification system. Both supervised and unsupervised treatment groups were given instructions to perform a new thick blood smear in case they presented with new symptoms (passive surveillance). All exams and positive malaria cases are reported to the national malaria surveillance system (SIVEP-Malaria). Recurrent episodes were assessed within 180 days from the beginning of anti-malarial treatment. No active surveillance was performed and, therefore, in the absence of symptoms, no additional thick blood smears were collected for the study. Quality control of the microscopic diagnosis followed the standard routine of the Amazonas State Surveillance Central Laboratory, and a percentage of negative smears and all the positive smears were revised by an experienced microscopist.

Data extracted from the SIVEP-Malaria system were merged into electronic forms (REDCap). Descriptive statistics were used for the analysis of the demographic variables. The Student’s t test was used to compare means while Fisher’s exact or the Chi-squared (*χ*^2^) test were used to compare proportions, as appropriate. Kaplan-Meier survival estimates were used to compare recurrences in the groups during the 180 days. All analyses were performed using the Stata program v.15 (Stata Corp, USA). This study was approved by the Ethics Review Board (ERB) at *Fundação de Medicina Tropical Dr Heitor Vieira Dourado* (CAEE: 18314019.5.0000.0005). Consenting patients signed an ICF and the ERB waived the ICF for those patients who did not consent since they followed the standard care procedure without supervision.

## Results

From November 20th, 2019 to November 3rd, 2020, 117 participants were included and finished the 180-day follow-up period. In all, 56 (47.8%) were randomized for the supervised treatment group and 61 (52.2%) made up the unsupervised routine treatment group. There were no significant differences between the characteristics of the groups at baseline (Table 1).

**Table 1:** Clinical and demographic characteristics of 117 participants at time of inclusion

Variable	Total	Supervised	Unsupervised
	*n* = 117	*n* = 56 (47.8%)	*n* = 61 (52.2%)
Age (±SD)	38.3 (14.4)	36.6 (14.3)	39.9 (14.6)
Gender (F)	41/117 (35.0%)	21/56 (37.5%)	20/61 (32.8%)
Education level			
Incomplete primary school	36/117 (30.8%)	14/56 (25.0%)	22/61 (36.1%)
Complete primary school	14/117 (12.0%)	4/56 (7.1%)	10/61 (16.4%)
Incomplete high school	29/117 (24.8%))	14/56 (25.0%)	15/61 (24.6%)
Complete high school	27/117 (23.1%)	15/56 (26.8%)	12/61 (19.7%)
Bachelor’s degree	11/117 (9.4%)	9/56 (16.1%)	2/61 (3.3%)
Area of residence			
Rural	98/117 (83.8%)	45/56 (80.4%)	53/61 (86.9%)
Urban	19/117 (16.2%)	11/56 (19.6%)	8/61 (13.1%)
Parasitaemia			
< +/2	20/117 (17.1%)	11/56 (19.6%)	9/61 (14.8%)
+/2	15/117 (12.8%)	9/56 (16.1%)	6/61 (9.8%)
+	26/117 (22.2%)	11/56 (19.6%)	15/61 (24.6%)
++	56/117 (47.9%)	25/56 (44.6%)	31/61 (50.8%)

*SD* standard deviation, *F* female

Parasitemia described by the Brazilian Ministry of Health: < +/2 = less than 40 parasites in the 100 fields examined, +/2 = 40 to 60 parasites in 100 microscopic fields, + = 1 parasite per field, ++ = 2 to 20 parasites per field, parasites/µL

**Fig. 1:**
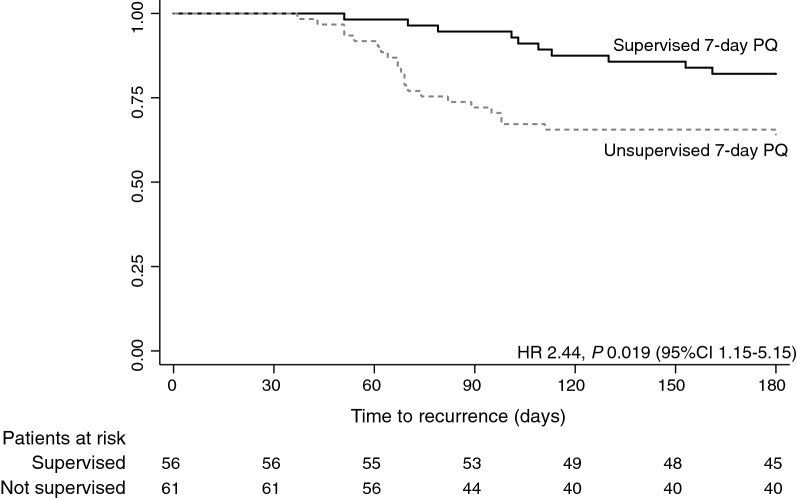
Time to first recurrence in the groups of supervised and unsupervised 7-day primaquine treatment in 180 days of follow-up. PQ, primaquine; HR, hazard ratio; CI, confidence interval

When comparing recurrences between groups, 32 (27 %) participants had at least 1 vivax malaria recurrence, with significant statistical difference between supervised and unsupervised treatment (18 % vs 36 %; *p *= 0.027) (Table 2). There was also a significant difference in the time to first recurrence (*p *= 0.04). Survival analysis showed a higher risk of recurrence in the unsupervised treatment group [Hazard Ratio 2.44, *p=*0.019 (95%CI 1.15–5.15)] when compared to supervised treatment (Fig. 1).

**Table 2:** Recurrences between supervised and unsupervised treatment groups with 180-day follow-up period

	Total	Supervised	Unsupervised	*P*
	*n* = 117	*n* = 56 (47.8%)	*n* = 61 (52.2%)	
Recurrence in 180 days (n/N)	32/117 (27%)	10/56 (18%)	22/61 (36%)	**0.027**
Number of recurrences (n/N)				0.07
1	18/32 (56%)	8/10 (80%)	10/22 (46%)	
≥ 2	14/32 (43.8%)	2/10 (20%)	12/22 (54%)	
Time to first recurrence (d) (n/N)				**0.04**
≤ 60	6/32 (19%)	1/10 (10%)	5/22 (23%)	
61–90	14/32 (44%)	2/10 (20%)	12/22 (54%)	
91–180	12/32 (37 %)	7/10 (70%)	5/22 (23%)	

The denominator for the number of recurrences and time to recurrence is the number of recurrences observed in each group

## Discussion

Results show that the unsupervised short PQ treatment presented an increased risk of recurrence compared to the supervised group and a greater proportion of patients in the unsupervised PQ group had multiple recurrences, which is an indication that the “bank” of hypnozoites persisted [14]. Since the middle 1990s, the shorter PQ regimen (7 days of 0.5 mg/kg/day) is used in Brazil to improve adherence, and the 14-day (0.25 mg/kg/day) course actually does not seem to be superior in preventing relapses [15]. However, data on compliance to the 7-day regimen is still scarce and has to deal with the methodological issues of randomizing a non-supervision group to serve as a comparator, in which the mere commitment to the study may increase drug intake, not reflecting therefore, the real-life situation. In order not to cause any distortion of reality, which could influence adherence, an unsupervised treatment group was used in our study, taking advantage of Zelen’s design, in which some patients were not included for consent and did not have their treatment supervised and, therefore, no intervention or contact with the participant happened throughout the study duration. The ERB permitted a waiver of the consent in this group, since it was understood that any consent process would bias the results. All randomized participants in the supervised group had home visits for drug administration from D1 to D7.

In Thailand and Papua New Guinea, a recurrence rate for *P. vivax* after treatment with CQ and PQ has been observed to reach up to 65% over 30 to 180 days of follow-up [16]. In the current study, we demonstrated that relapses occurred between 42 and 180 days, possibly the high level of relapses in the group of unsupervised participants was due to non-adherence. In another study in the Brazilian Amazon, in a portion of recurrent *P. vivax* malaria episodes within 90 days of a recurrence of a previous episode, the rate was similar (29.44%) [12]. Many studies have shown that there are a number of problems in the treatment of *P. vivax* malaria, one of which is the precariousness of the dispensing system and inadequate storage conditions. Several other factors can contribute to the increase in recurrence rates in the Amazon region, including genetic factors, e.g., abnormal CYP2D6 activity [17].

The number of *P. vivax* patients who experienced recurrence in this study raises the hypothesis that recurrence in vivax cases was higher due to improper treatment of these cases [18], considering that participants in each group were randomized. In the Brazilian Amazon region, treatment is not supervised and there are few studies that discuss the importance of adherence and the often-precarious conditions of dispensing and storing medications can contribute negatively [19]. In the state of Pará, Brazil, one study reported that the relative risk of parasitic resurgence was 3.04 times higher in patients that did not adequately adhere to treatment, and also reported that adherence frequency was 86.4% (81.7%–90.1%) [20].

Non-adherence to treatment affects the health of patients and is one of the main factors of therapeutic failure that directly impacts the control of the disease and places a socioeconomic burden on health systems [21]. In Brazil, several factors are related to non-adherence to treatment, such as the rapid disappearance of symptoms after the start of treatment which causes patients to abandon treatment, the adverse effects of medications, inadequate prescriptions or dispensing, as well as the difficulty some patients have in understanding instructions [13, 19]. Information on non-adherence to the current anti-malarial treatment is essential for interventions aimed at reducing therapeutic failure and further recurrences. However, with the possibility of introducing tafenoquine, the 8-aminoquinoline given as a single dose, with similar efficacy to 14-day PQ regimen [22], there is a concern that the high levels of efficacy observed in clinical trials may not be repeated in the real life [23].

The major limitations of the study include the small sample size, which in the future should be increased to include other endemic areas in Brazil, and increase national representativeness, and the fact that no compliance could be estimated in those under 18 years of age. There is also an underestimation of asymptomatic relapses, as no active microscopic surveillance was performed, and, regarding time of illness, unfortunately, since the outcome of interest was recurrence, the patients were not followed up in regards to cure or discharge, thus no data is available for incidence density (number of recurrences/person year).

These preliminary data, from a municipality in the Brazilian Amazon, may be used as a first reliable reference, based on real life data, since it gives an indication to which extent the lack of compliance to the 7-day PQ regimen in the treatment of vivax malaria affects recurrences up to day 180. Non-supervision more than doubles such risk, which might be a bottleneck for any malaria elimination programme.

## Conclusions

Treatment supervision provides an additional valuable tool for the elimination of vivax malaria in this scenario. Future studies should be multicentre type studies, in order to assess different environments and patient profiles. Additionally, the perception of failure of or adherence to treatment can be assessed by qualitative studies, in order to understand the local factors associated with recurrences.

## Data Availability

Datasets from the current study are available upon reasonable request to the corresponding author.

## References

[1] Ministério da Saúde. Secretaria de Vigilância em Saúde. Guia prático de Tratamento da Malária no Brasil. Departamento de Vigilância Epidemiológica, Brasília; 2020. https://bvsms.saude.gov.br/bvs/publicacoes/guia_pratico_malaria.pdf. Accessed 10 Jan 2021.

[2] Ministério da Saúde. Secretaria de Vigilância em Saúde. Guia para gestão Local do Controle da Malária: Controle Vetorial. Brasília; 2008. http://bvsms.saude.gov.br/bvs/publicacoes/guia_gestao_local_controle_vetorial.pdf. Accessed 12 Jan 2021.

[3] Commons RJ, Simpson JA, Thriemer K, Humphreys GS, Abreha T, Alemu SG (2018). The effect of chloroquine dose and primaquine on *Plasmodium vivax* recurrence: a WorldWide Antimalarial Resistance Network systematic review and individual patient pooled meta-analysis. Lancet Infect Dis.

[4] Vieira GD, Gim KNM, Zaqueo GM, Alves TC, Katsuragawa TH, Basano SA (2014). Reduction of incidence and relapse or recrudescence cases of malaria in the western region of the Brazilian Amazon. J Infect Dev Ctries.

[5] Vitor-Silva S, Siqueira AM, de Souza Sampaio V, Guinovart C, Reyes-Lecca RC, de Melo GC (2016). Declining malaria transmission in rural Amazon: changing epidemiology and challenges to achieve elimination. Malar J.

[6] Duarte EC, Gyorkos TW (2003). Self-reported compliance with last malaria treatment and occurrence of malaria during follow-up in a Brazilian Amazon population. Trop Med Int Health.

[7] Yeung S, White NJ (2005). How do patients use antimalarial drugs? A review of the evidence. Trop Med Int Health.

[8] Bruxvoort K, Goodman C, Kachur SP, Schellenberg D (2014). How patients take malaria treatment: a systematic review of the literature on adherence to antimalarial drugs. PLoS One.

[9] Hill DR, Baird JK, Parise ME, Lewis LS, Ryan ET, Magill AJ (2006). Primaquine: report from CDC expert meeting on malaria chemoprophylaxis. Am J Trop Med Hyg.

[10] Silva RSU, Pinto AYN, Calvosa VSP, Souza JM (2003). Esquemas terapêuticos encurtados para o tratamento de malária por *Plasmodium vivax*. Rev Soc Bras Med Trop.

[11] White NJ (2011). Determinants of relapse periodicity in *Plasmodium vivax* malaria. Malar J.

[12] Silva VS, Siqueira AM, de Souza Sampaio V, Guinovart C, Reyes-Lecca RC, de Melo GC (2016). Declining malaria transmission in rural Amazon: changing epidemiology and challenges to achieve elimination. Malar J.

[13] Pereira EA, Ishikawa EA, Fontes CJ (2011). Adherence to *Plasmodium vivax* malaria treatment in the Brazilian Amazon Region. Malar J.

[14] Ashley AA, Phyo AP, Carrara VL, Tun KM, Nosten F, Smithuis F (2019). *Plasmodium vivax* Relapse rates following *Plasmodium falciparum* malaria reflect previous transmission intensity. J Infect Dis.

[15] Daher A, Silva JCAL, Stevens A, Marchesini P, Fontes CJ, ter Kuile FO (2019). Evaluation of *Plasmodium vivax* malaria recurrence in Brazil. Malar J.

[16] Betuela I, Rosanas-Urgell A, Kiniboro B, Stanisic DI, Samol L, de Lazzari E (2012). Relapses contribute significantly to the risk of *Plasmodium vivax* infection and disease in Papua New Guinean children 1–5 years of age. J Infect Dis.

[17] Pukrittayakamee S, Chantra A, Simpson JA, Vanijanonta S, Clemens R, Looareesuwan S (2000). Therapeutic responses to different antimalarial drugs in vivax malaria. Antimicrob Agents Chemother.

[18] Brasil LW, Rodrigues-Soares F, Santoro AB, Almeida ACG, Kühn A, Ramasawmy R (2018). CYP2D6 activity and the risk of recurrence of *Plasmodium vivax* malaria in the Brazilian Amazon: a prospective cohort study. Malar J.

[19] Prasad RN, Virk KJ, Sharma VP (1991). Relapse/reinfection patterns of *Plasmodium vivax* infection: a four year study. Southeast Asian J Trop Med Public Health.

[20] Almeida ED, Vieira JLF (2016). Factors associated with non-adherence to the treatment of vivax malaria in a rural community from the Brazilian Amazon Basin. Rev Soc Bras Med Trop.

[21] Simões LR, Alves ER, Ribatski-Silva D, Gomes LT, Nery AF, Fontes CJF (2014). Fatores associados às recidivas de malária causada por *Plasmodium vivax* no Município de Porto Velho, Rondônia, Brasil, 2009. Cad Saúde Pública.

[22] Llanos-Cuentas A, Lacerda MV, Rueangweerayut R, Krudsood S, Gupta SK, Kochar SK (2014). Tafenoquine plus chloroquine for the treatment and relapse prevention of Plasmodium vivax malaria (DETECTIVE): a multicentre, double-blind, randomised, phase 2b dose-selection study. Lancet.

[23] Val F, Costa FT, King L, Brito-Sousa JD, Bassat Q, Monteiro WM (2019). Tafenoquine for the prophylaxis, treatment and elimination of malaria: eagerness must meet prudence. Future Microbiol.

